# Evaluation of the Content of Polyphenols, Antioxidant Activity and Physicochemical Properties of Tortillas Added with Bambara Groundnut Flour

**DOI:** 10.3390/molecules25133035

**Published:** 2020-07-03

**Authors:** Mpho Edward Mashau, Tumelo Mabodze, Ompilela Justice Tshiakhatho, Henry Silungwe, Shonisani Eugenia Ramashia

**Affiliations:** Department of Food Science and Technology, School of Agriculture, University of Venda, Private Bag X5050, Thohoyandou 0950, South Africa; tumelo.mabodze@gmail.com (T.M.); tshiakhathooj@gmail.com (O.J.T.); henry.silungwe@univen.ac.za (H.S.); shonisani.ramashia@univen.ac.za (S.E.R.)

**Keywords:** tortillas, Bambara groundnut, maize (*masa*), proximate composition, thermal properties and bioactive compounds

## Abstract

The effect of substituting maize (*masa*) flour with Bambara groundnut flour in tortillas production was investigated. Thermal, antioxidant, physicochemical properties, degree of puffing and rollability of flour and tortillas were determined. Tortillas were produced from maize and Bambara Groundnut (BGN) flours at the ratio of 100:0, 95:5, 90:10, 85:15 and 80:20, respectively. Compositing maize with BGN flour showed an improvement on the proximate composition of maize flour and tortillas; however, carbohydrate content of tortillas significantly decreased with the addition of BGN in blends from 77.07 to 55.22. The temperatures of gelatinisation such as onset temperature (T_o_) of flour blends increased from 57.50 to 71.95 °C, peak temperature (T_p_) from 74.94 to 76.74 °C and the end temperature (T_e_) from 81.72 to 91.58 °C. Composite flours and tortillas had higher values of polyphenolic compounds and antioxidant activities than the control sample. Textural properties of control tortillas were higher than that of composite tortillas. Increase in the levels of BGN flour improved the weight and thickness of tortillas. However, diameter and spread ratio decreased. Degree of puffing and rollability of tortillas increased with the incorporation levels of BGN flour.

## 1. Introduction

Consumers always demand food products that contain high nutritional value as well as furnishing extra health benefits. Therefore, there is a need for the food industry to develop new products with added potential health benefits. Tortilla is a flat unfermented bread prepared either from maize or wheat and has been widely consumed as the staple bakery food in Mexico and some Central America countries for centuries [1]. However, it is known that zein which is the main protein fraction in maize has deficiencies of lysine and tryptophan. Moreover, tortillas lack major vitamins and minerals. This can be alleviated by incorporating health-promoting compounds to the tortillas as is frequently done in various foods [2,3]. Tortillas can be used as a vehicle to reduce protein energy malnutrition if the flour is supplemented with products such as Bambara groundnut [4]. Different studies have been conducted to alter the taste, texture and nutritional value of tortillas whereby the dough was incorporated with other flours. For example, tortilla is fortified with *Moringa oleífera* flour, amaranth flour, unripe banana and cassava flours, flaxseed flour and iron and other micronutrients [5,6,7].

Bambara groundnut (*Vigna subterranae* (L). Verdc) is a legume crop originally from Africa. Bambara groundnut (BGN) is among the most adaptable plants and it withstands harsh conditions better than many crops [8]. It has received much attention for uses in various types of food systems due to its extensive distribution globally and high amount of protein content [9]. BGN is a well-balanced food because it is also rich in iron compared to most food legumes, and good quality protein and it is a good source of fibre, potassium and calcium [10]. It has more essential amino acids compared to other leguminous crops. In terms of protein score comparability, BGN has 74% and 64% higher than soya bean and cowpea, respectively. However, it is well known that all legumes including BGN are deficient in sulphur containing amino acids. Therefore, mixing BGN with a staple cereal grain such as maize is a desirable nutritional approach since maize has higher amounts of cysteine and methionine deficient in legumes [11]. Although BGN produces food with better nutrition, it is still one of the neglected crops in terms of research and development, becoming less important when compared to other well-known legumes and cereal crops and slowly losing its diversification and associated indigenous knowledge [12,13]. Due to the growing demand for consistent quality of nutraceuticals food and supplements, there is a need to promote indigenous crops since it appears as a possible way of dealing with malnutrition in developing countries.

When BGN flour is mixed with maize flour it can modify the nutritional value of the tortillas. This is important since food industries have the opportunity of utilising BGN flour as a functional ingredient and expand its usage during the processing of cookies, pasta, snacks and bread. However, information on the utilisation of BGN flour to produce fortified food with improved nutritional value remains inadequate. Therefore, this study evaluated the effect of BGN flour on the polyphenols, antioxidant activity and physicochemical properties of maize flour and tortillas. We hope that the results obtained will contribute to the general use of BGN flour in value added products.

## 2. Result and Discussion

### 2.1. Thermal Properties of Composite Flour Blends

Table 1 presents the thermal properties of control and maize–Bambara groundnut (BGN) flour blends. The onset temperature ranged from 57.5 to 71.95 °C. There was significant difference (*p* < 0.05) among the onset temperature of all flour blends and they showed an increasing trend with an increase of BGN flour. The inclusion of BGN flour at all levels resulted in an improved onset temperature of maize-BGN flour blends. The presence of mucilage in the BGN flour might have contributed to the increase of onset temperature of maize-BGN flour blends. Mucilage contains protein and complex polysaccharides that could compete with starch for moisture leading to higher onset starch gelatinisation temperatures in the flour blends [14]. Other components such as protein, fat, fibre and minerals might also have contributed to this behaviour since BGN has high nutritive value containing 53–65% carbohydrate, about 20% protein, 6.5% fat, 6.1% fibre, 3.4% ash, as well as appreciable amounts of calcium, iron, sodium and potassium [15].

Peak temperature ranged from 74.94 to 78.45 °C, control and BGN-maize flour blends showed significant difference at *p* < 0.05. The peak temperature obtained follows the same trend reported for other flour such as cowpea (67–78 °C) [16]. However, the results are lower than the one obtained by Sirivongpaisal [17] and this could be due to the difference on the granular structures of BGN flour compared to the composite flour [18]. Moreover, low damaged starch content of BGN flour might also have contributed to the increase of peak and onset temperatures of BGN-maize flour blends since it allowed the formation of strong bonds between starch granules and water molecules. These differences in peak and onset temperature results between control and composite flours may also be due to water competition phenomena between starch and other components.

The end temperature ranged from 81.72 to 91.58 °C and there was variation in the values. Higher gelatinisation end temperature in control sample is attributed to its larger granule size. Larger granules tend to have poorer hydration and swelling capacity than smaller granules. On the other hand, the low end temperature in BGN-maize flour blends could be due to large amounts of short amylopectin chains present in the starch of the flour blends [19]. The enthalpy of gelatinisation ranged from 5.57 to 14.81 J/g and there was variation in the values of control and composite flour blends. BGN_4_ had the highest value of gelatinisation enthalpy and this shows that BGN starch is more stable during baking and it increased the thermal stability of flour blends [20].

Gelatinisation enthalpy indicates the thermal stability of starch. Values of ΔH at different levels of composite flour blends might be attributed to the size of starch granules and amylopectin ratio. BGN flour interacted with the matrix of maize flour which caused a release of water and matric hardening resulting in a higher gelatinisation temperature. Higher values of gelatinisation enthalpy of composite flours suggest mild processing conditions because thermal treatment produces starch gelatinisation with a higher degree of disorder. In addition, higher values of gelatinisation enthalpy will result in tortillas with denser cell walls that will require higher force to rupture [21]. Texture changes of high-starch products are mainly related to starch gelatinisation and retrogradation. The rate of dehydration and starch retrogradation are the factors that contribute to the hardening of tortillas, an undesirable characteristic since consumers prefer soft tortillas [22]. The values of the energy flow of BGN flour are in line with report by Kaptso et al. [16].

### 2.2. Proximate Composition of Maize-Bambara Groundnut Flour Blends and Tortillas

The proximate composition of maize flour and tortillas added with BGN flour is shown in Table 2. The moisture content of the composite flour blends showed a significant increase with the inclusion levels of BGN flour, varying from 3.69 (control) to 4.39% (BGN_4_). The low moisture content of control and composite flour blends is a good indication of microbiological stability of the flour during storage and can also lead to a reduction in the stalking tendency in baked goods [23]. The moisture content of composite flours fall within the recommended range of 12.0–13.0% for stability of flour. These results were lower than the moisture content of 11.3–11.6% reported by Kaptso et al. [16]. The moisture content of flours was lower than that of tortillas and this may be due to the amount of water added during dough development to produce tortillas. The moisture content of tortillas ranged from 14.93 (control) to 29.86% (BGN_4_) and there was a significant difference among all tortillas samples (*p* < 0.05). The control tortillas had the lowest moisture content than the composite tortillas. The significant increase in the moisture content of tortillas with the increase of BGN flour could be attributed to the nature of BGN which increased the overall water holding capacity of the tortillas. The moisture values are within the range (35–50%) reported for tortillas, depending on the conditions of nixtamalisation and variety of maize. However, higher moisture content of between 45%-50% makes the tortillas very susceptible to microbial spoilage. Moulds and yeasts become visible on tortillas stored at room temperature after 48 h but this can be prevented by keeping tortillas in an alkaline pH (>9) [24]. These results are similar with the increasing trend of moisture content in composite tortillas [5].

The ash content of flour ranged from 1.23 (control) to 3.08% (BGN_4_). The ash content was related to the amount of BGN flour added and this shows that BGN is rich in minerals than maize. The ash content of tortillas varied from 1.04 to 1.63%. The control sample was significantly different (*p* < 0.05) from all the samples and it had the smallest quantity of ash (1.04%) compared with composite tortillas. However, the ash content of tortillas was lower than that of flour blends and this could have been caused by heat during baking of tortillas since some minerals such as potassium, copper and zinc are decreased during cooking/high temperatures [25].

There was a significant increase in the protein content of composite flour samples from 3.32 (control) to 7.24% (BGN_4_). The increase in the protein content of composite flours with the quantity of BGN flour added is due to that BGN is a complete balanced food high in protein content rich with lysine and methionine while maize lacks lysine [26]. The protein content of tortillas ranged from 3.14 (control) to 6.33% (BGN_4_) and there was significant difference (*p* < 0.05) among all the tortillas samples except BGN_2_ and BGN_3_. The heat during the baking process induces oxidation through the generation of radicals [27]. The incorporation of BGN flour increased amounts of amino acids in tortillas, which are susceptible to protein oxidation and this might have contributed to the observed results in BGN_2_ and BGN_3._ There was an increasing trend on the protein content of tortillas with the increase in the inclusion levels of BGN flour. There was a slight decrease on the protein content of the composite tortillas when compared with the flour blends. This might be due to the heat during the baking of tortillas because amino acid chains are broken down and protein denatures at high temperatures. Similar results were reported by Vazquez-Rodrigues et al. [28] on tortillas added with bean and amaranths flour.

The fat content of composite flour blends ranged from 1.74 to 2.95% with control having the lowest value. The fat content of the composite flour blends increased with the incorporation of BGN flour in the blends. The fat content of tortillas ranged from 1.73% (control) to 2.93% (BGN_4_). The high fat content in composite tortillas could be attributed to that BGN comparatively has more fat than maize [29]. Diets with high fat content show significant contribution to the energy needs of humans because one gram of fat or oil will yield about 368 kJ/gkcal of energy when oxidised in the body [30]. There was minor difference on the fat content of composite flours and tortillas and this may be because the thermal processing of tortillas did not affect the fat content quantity but the quality. Similar increase of fat content of composite tortillas was observed by Páramo-Calderóna et al. [5] on tortillas fortified with *Moringa oleifera* flour.

The fibre content of the flour blends varied from 2.03 to 3.53% and there was a significant difference (*p* < 0.05) among all the samples except between control and BGN_1_. The fibre content of tortillas ranged from 2.09 to 4.03% with the control sample having the lowest value. The fibre content of the flour blends was slightly higher than that of tortillas and this could be due to the heat during processing of tortillas as it has been reported that cooking/heating reduces the fibre content of food [31]. However, the availability of fibre in food is known to have beneficial effect since it has physiological effects in the gastrointestinal tract [32].

The carbohydrate content of composite flours decreased from 88.00 to 78.18% with the addition of BGN flour in maize flour. The carbohydrate content of tortillas decreased from 77.07–55.22% with the amount of BGN flour added to maize flour, all the samples differed significantly at *p* < 0.05. Carbohydrate content of tortillas decreased slightly compared to the composite flour blends, the changes observed may possibly be due to the heat destruction of nutrients such as protein and fibre during baking of tortillas [33]. Moreover, carbohydrates tend to breakdown into simple sugars when they are exposed to heat. This is expected since starch is degraded by enzymes and transformed into soluble sugars during cooking processes [34]. At high temperature (110 °C–140 °C) to which the outer part of the dough is exposed; starch degrades to dextrin, mono and disaccharide. The exhibited behaviour in the composite tortillas might be attributed to differences in the rate of starch hydrolysis due to presence of fibre, possible presence of natural enzyme inhibitors during analysis and inherent differences in starch structure and composition [35,36]. This might also explain high values of moisture, ash, protein, fat and fibre in fortified tortillas.

### 2.3. Polyphenolic Compounds and Antioxidant Activities of Maize-Bambara Groundnut Flour Blends and Tortillas

The analysed data of total phenolic content (TPC) of flour blends and tortillas is presented in Table 3. The TPC values ranged from 43.82 ± 2.00 to 72.05 ± 4.22 mg GAE/g and 46.61 ± 2.10 to 79.31 ± 5.60 mg GAE/g for maize-BGN flour blends and tortillas, respectively. However, the TPC content increased slightly in the tortillas compared to flour blends. The slight increase in TPC of tortillas could be related to the liberation of more bound phenolic during baking since cellular constituents are broken down during thermal treatment [37]. A similar trend of increase in TPC of composite flour blends was reported by Abdel-Gawad et al. [38]. In addition, the formation of Maillard reaction products with the structure of phenolic compounds during baking could at least partially explain the increase of TPC in composite tortillas [39]. The results obtained show that BGN is a greater source of TPC and can be used to composite cereal based products to enhance their bioactive compounds to ensure better health benefits of the consumers. The inactivation of oxidative enzymes by heat during baking could also explain the increase of the TPC in the tortillas. Shahidi and Wanasundara [40] proposed that the action of oxidative enzymes such as polyphenol oxidase and peroxidases contribute to greater losses of phenolic compounds during the thermal processing of food. Similar results of increase in TPC of composite tortillas were reported by Páramo-Calderóna et al. [5] on tortillas added with *Moringa oleifera* flour and Hernandez-Martinez et al. [41] on tortillas made from pigmented maize.

The total flavonoids content (TFC) of both composite flour blends and tortillas varied significantly (*p* < 0.05) except BGN_1_ and BGN_2_ of tortillas which were not significantly different. TFC values ranged from 0.14 to 0.588 mg CE/g and 0.03 to 0.47 mg CE/g for both maize-BGN flour blends and tortillas, respectively. Both composite flour blends and tortillas showed an increasing trend with the increase in BGN flour inclusion. However, there was a decrease in the flavonoids content of composite tortillas compared to flour blends and this could be due to the breaking down of flavonoids and glycosides extraction by heat and steam during baking [42]. Such processes were reported in other research where flavonoid-containing plant material was thermally processed. Flavonoids are heat sensitive and heating at 75 °C can destroy enzyme activity and block the synthesis pathway of flavonoids [43]. This may be the reason why the TFC of BGN_1_ and BGN_2_ tortillas was also similar. Moreover, the decrease in TFC of composite tortillas might be due epolymerisation of polyphenols and decarboxylation of phenolic acids that occur during thermal treatment [44]. However, the development of antioxidant compounds such as hydroxymethylfurfuraldehyde which is a product of Maillard reaction product contributes to the stability of flavonoids during the baking process [45]. The results obtained agree with those reported by Li et al. [46] that thermal treatment decreases the flavonoids content of grains.

The antioxidant activity of maize flour and tortillas incorporated with BGN flour at various levels was evaluated by DPPH and FRAP methods (Table 3). The inclusion of BGN flour at different levels in the maize-BGN flour blends and tortillas increased the DPPH activity from 60.70 to 90.99% and 43.09 to 80.54% for composite flour blends and tortillas, respectively. These results demonstrated that DPPH activity differed significantly at (*p* < 0.05) in control and composite flour blends and tortillas. Control flour and tortillas had lower radical scavenging ability than that added with BGN flour. The increase in DPPH activity of composite blend flours and tortillas is due to the higher radical scavenging activity of BGN flour. The development of melanoidins during baking further increased the radical scavenging ability of composite tortillas. In addition, the increase in DPPH activity of composite tortillas could be attributed to the heat during baking which contributed to bioactive compounds such as ferulic acid from matric interacting with some antioxidant substances that were dissolved in water during dough formation [47]. A higher DPPH value is related to stronger antioxidant activity and a lower value is associated with a weaker antioxidant activity.

The Ferric Reducing Ability of Plasma (FRAP) activity of the composite flour blends varied from 0.071 to 0.156 mg GAE/g and for tortillas ranged from 0.106 to 0.183 mg GAE/g, respectively. The control flour and tortillas had lower FRAP activity than that added with BGN flour. The inclusion of BGN flour increased the FRAP activity of maize-BGN flour blends and tortillas.

The increment of antioxidant activity of flours and tortillas is associated with the increase of TPC when incorporating BGN flour. Therefore, the BGN flour could be used as an alternative source of antioxidants that would enhance the nutritional characteristics of the tortillas. These findings suggest that consumption of BGN complemented product could offer some health benefits especially thermal processed products because they contain a lot of antioxidants reducing power. Sharma and Gujral [48] reported similar results of increase in antioxidant activity of composite flour from wheat and barley flours. These results are in line with those by Hernandez-Martinez et al. [41] which showed an increase in antioxidant activity of tortillas prepared from similar varieties of blue and purple maize. These researchers concluded that the increase in antioxidant of tortillas was due to availability of anthocyanins and polyphenols in maize flour. They also indicated that flavonoids such as kaempferol and morin also contributed to the increase in the antioxidant activity of fortified tortillas.

### 2.4. Texture Profile of Tortillas Added with Bambara Groundnut Flour

Table 4 shows the texture profile analysis of control and tortillas added with BGN flour. A significant difference (*p* ≤ 0.05) was observed for control and composite tortillas. The results show that the control sample had a higher hardness, springiness, gumminess, cohesiveness and chewiness values than the tortillas containing BGN flour. The observed difference could be attributed to the fact that the control tortillas contain high starch (amylose and amylopectin) content. A three dimensional network is created by gelation of amylose right after baking. Amylose associates to form an insoluble network that causes a flexible gel structure in maize tortillas [49]. In addition, starch retrogradation could also have contributed to high hardness, cohesiveness, springiness, gumminess and chewiness of the control tortillas. Hardness is related to starch retrogradation which starts immediately after cooling the tortillas. The control tortillas sample had a faster retrogradation process because of its linear, highly polar formation, which reduced its ability to hold water and this resulted in partial shrinkage of the starch. The inclusion of BGN flour in tortillas altered this process due to its high protein content, the water holding capacity increased and this prevented the tortillas from becoming rigid and hard [50]. The hardness value (8.99 N) for the control tortillas was very similar with 8.82 N for BGN_1_. These values are higher compared to that reported by Chel-Guerrero et al. [51] (3.16–4.39 N) in maize tortillas. The variety of maize used, the moisture of the dough as well as higher gelatinisation enthalpy of the flours can be attributed to the difference. However, the values are similar to that reported by Páramo-Calderóna et al. [5] (6.79–9.38 N) in tortillas added with *Moringa oleifera* flour.

In addition, control tortillas had the highest cohesiveness value. Two important factors could be attributed to this behaviour, the hydration ability by the inclusion of BGN flour as well as its fibre content, which perhaps reduced the association between the water and the constituents of the flour dough. Results showed that BGN flour significantly improved the textural properties of the tortillas, as observed by the decrease in hardness, springiness, cohesiveness, gumminess and chewiness of the tortillas. Reyes-Vega et al. [52] reported that the textural properties of the corn tortillas can be characterised by principal parameters such as hardness, springiness and cohesiveness. These findings are consistent with those by Reyes-Vega et al. [51] and Arambula-Villa et al. [53] on the textural analysis of tortillas.

### 2.5. Physical Properties of Tortillas Added with Bambara Groundnut Flour

The physical properties of tortillas prepared from maize-BGN flour blends are presented in Table 5. Weight, diameter, thickness and spread ratio of the tortillas were significantly different at *p* ≤ 0.05. Tortillas prepared only with maize flour were less heavy than those prepared from BGN and maize flour blends. The weight increased as the more BGN flour was added to the formulation. The weight ranged from 35.93 to 39.26 g. The diameter of the composite tortillas was significantly lower than that of the control tortillas ranging from 13.07 (BGN_4_) to 14.60 cm (control).

The thickness of the tortillas ranged from 2.17 (control) to 3.07 mm (BGN_4_). The decrease in diameter might have influenced the increase in thickness of the tortillas. Baljeet et al. [54] found that the thickness of cookies increases as the diameter decreases. Similar results were observed by Hussein et al. [55], whereby weight and thickness of tortillas increased as more triticale flour was added to the maize flour. The increase in the weight and diameter of tortillas was attributed to the higher protein content found in triticale.

In this study, as more BGN flour was added, the weight and diameter also increased because of the reasonable amount of protein content in BGN flour. It can be concluded that the thicker the tortillas, the lower the diameter of the tortillas. The incorporation of BGN flour to the formulation resulted in the decrease of the spread ratio of the tortillas. The low values of the spread ratios of the tortillas were due to the aggregates formed by the flour samples with higher numbers of hydrophilic sites available to compete for free water in the dough [56].

### 2.6. Degree of Puffing and Rollability of Tortillas Added with Bambara Groundnut Flour

**Figure 1:**
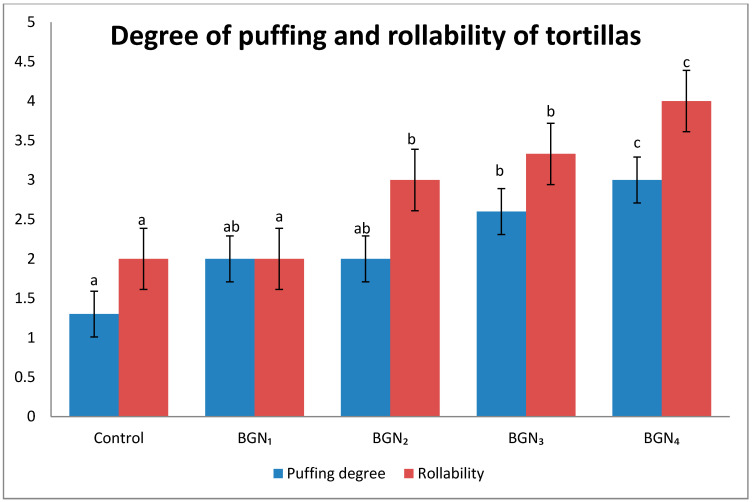
Quality properties of tortillas added with Bambara groundnut flour. BGN_1_, BGN_2_, BGN_3_ and BGN_4_ refer to the tortillas prepared from Bambara groundnut and maize flour (BGN: maize) in the percentages of 5:95, 10:90, 15:85 and 20:80 and maize flour was used as control. Different alphabets on bars indicate statistically different effect (*p* ≤ 0.05).

The results of puffing degree and rollability of tortillas are presented in Figure 1. Results show that the control tortillas had the lowest value for puffing degree (1.4). However, both control tortillas and BGN_1_ sample had the lowest values of rollability (2.0). The puffing degree and rollability increased as more BGN flour was added to the formulation and full puffing of the tortillas was observed in BGN_4_ sample. The observed variations in puffing degree might be attributed to the fact that puffing takes place during the baking of tortillas owing to the creation and aggregation of steam between the two layers of the tortillas [57]. Initially, when puffing takes place, there is coalascion of the two dissociated layers of the tortillas to form one large bubble. However, the bubble only takes place when cracks do not form on the crust of the tortillas which will enable the steam to escape, causing the bubble to collapse [58].

Rollability of the composite tortillas increased as more BGN flour was added to the formulation although the cohesiveness of the tortillas added with BGN was low when compared with the control tortillas. Basic ingredients such as water and fat are considered to be dough conditioners because they directly impact the way the dough is formed and its final structure; this improves the softness and rollability of tortillas [5]. This might explain high rollability values of tortillas added with BGN flour even though their cohesiveness values were very low. Rollability measures cracking and breakage of tortillas. The variations in the results obtained might be attributed to that immediately after baking, tortillas are soft and flexible, easy to roll or fold without causing cracks. Over baking damages the starch protein which leads to reduced rollability of tortillas. Moreover, when tortillas become stale, they experience changes that make them more firm, hard and crack easily during the rolling or folding process [59]. Similar results were observed by Moreno-Castro et al. [60] whereby the rollability increased with the substitution of maize with flour that contains higher protein content, triticale and chia seed flour.

## 3. Materials and Methods

### 3.1. Material

Creamy white BGN, shortening and salt were obtained from a local market. Commercial “*masa*” (corn dough) was obtained from Azteca Mexican Products CC, Midrand, Gauteng Province, South Africa. All chemicals were purchased from Merck, Centurion, South Africa, and were of analytical grade.

### 3.2. Bambara Groundnut Flour Preparation Flour

Approximately 2 kg of Bambara groundnut (BGN) was sorted to remove foreign materials, damaged and spoiled groundnuts. They were soaked in 5 L of tap water (1:4 *w*/*v*) for 12 h at room temperature. BGN were manually de-hulled and oven dried at 60 °C for 12 h. A disc attrition mill (Retsch KG 5657, Retsch GmbH, Haan, Germany) was used to mill BGN into flour and sifted using a 355 μm sieve.

### 3.3. Blend Formulation

Composite flour was prepared by mixing BGN with maize flours in various ratios (BGN_1_-5:95, BGN_2_-10:90, BGN_3_-15:85 and BGN_4_-20:80). Composite flours were prepared to make dough and were thoroughly mixed to obtain uniformity. The composite flour samples were sealed in polyethylene bags and stored at 4 °C for further use.

### 3.4. Tortillas Preparation

The composite dough prepared from maize and BGN flours was used to produce ratios of 5, 10, 15 and 20% (*w*/*w*, dry basis). This was followed by adding water for “*masa*” formation to make tortillas. The dough was moulded into 50 g round pieces. The pieces were pressure pressed into 4 mm thick tortillas. A hot plate with shortenings was preheated to 250 °C and the tortillas were baked for 30 s on the first side, overturned and then baked for another 40 s on the second side, flipped and baked for another 10 s on the first side. A rack was used to cool the baked tortillas for 1 min and they were packaged in polyethylene bags and stored in a desiccator for subsequent analysis. The tortillas made from 100% maize dough were used as control.

### 3.5. Thermal Properties of Composite Flour Blends

The gelatinisation behaviour of composite flour blends from maize and BGN were measured as described by Sun et al. [61] using differential scanning calorimeter (DSC, DSC 4000, Perkin-Elmer, Shelton, CT, USA). Briefly, 20% flour suspension was prepared and 15 mg was transferred to a sample cell. All flour samples were allowed to equilibrate for 1 h before analysis. An empty aluminium pan was used as standard reference. Calorimeter scan conditions were set as follows: A heating rate of 10 °C/min was used to heat flour samples from 25 to 120 °C. Gelatinisation parameters measured were: T_o_ (onset temperature), T_p_ (peak temperature), T_e_ (end temperature) and ΔH (enthalpy of gelatinisation). Samples were analysed in triplicates and the obtained data was calculated using Pyris software (Perkin-Elmer, Shelton, CT, USA).

### 3.6. Proximate Composition of Composite Flours and Tortillas

Composite flour samples were analysed for proximate composition: moisture, ash, protein, fat and crude fibre were determined using hot air oven, Muffle furnace, micro-Kjedahl Nx6.25, soxhlet extraction and Fibre Tech following the method of Association of Official Analytical Chemists [62]. Carbohydrate content was determined from the percentage difference of the other proximate indexes as follows; %carbohydrate = 100 − %fat − %protein − %moisture −%ash − %fibre. All flour blends and tortillas samples were tested in triplicate.

### 3.7. Phenolic Compounds and Antioxidant Activity of Flour Blends and Tortillas

#### 3.7.1. Extract Preparation

Samples were extracted with acidified methanol (1 mL of HCl, 88 mL of methanol and 11 mL of distilled water). Approximately one gram of sample was added to 5 mL of acidified methanol at room temperature for 2 h in a water bath (Model no: WBH 601 Labcon) with constant shaking at every 10 min. The extract was separated in a centrifuge for 15 min at 4500 rpm. Supernatant was then collected.

#### 3.7.2. Determination of Total Phenolic Content

Folin–Ciocalteu colorimetric method was used to determine the total polyphenols of flour blends and tortillas. Briefly, 0.2 g of flour blends or tortillas was weighed and mixed with 2 mL of acetone. The mixture was incubated for a period of 1 h (shaking occasionally at 25 ± 2 °C). The mixture was centrifuged at a speed of 6000 rpm for 5 min at a temperature of 4 °C. About 109 µL of Folin Ciocalteu solution was added to 9 µL of a centrifuged sample placed in a microplate. Approximately 180 µL of Na_2_CO (7.5% concentration) was added to the mixture. Aluminium foil was used to cover the mixture before incubation at 50 °C for a period of 5 min. With the use of UV spectrophotometer microplate reader (Zenyth 200rt Biochrom, Cambridge, UK), the absorbance of the samples was read at 760 nm. The solvent used for extraction was acetone and the standard phenol compound used was Gallic acid. The results obtained were recorded as gallic acid equivalents from calibration curve [63].

#### 3.7.3. Determination of Total Flavonoids Content

The total flavonoid content of samples was determined using a colorimetric method with few modifications. Approximately 0.5 mL aliquots extracts were deposited into 15 mL conical tube which comprised 4 mL distilled water with 0.15 mL of ${\mathrm{NaNO}}_{2}$ (5% sodium nitrate). After 5 min, solution of 4 mL of distilled water and 5% of ${\mathrm{AlCl}}_{3}$ was added. The solution was allowed to stand for 5 min after the addition of 1 mL of NaOH. Afterwards, the solution was thoroughly mixed and 1 mL of NaOH was added again before the solution could stand for about 15 min. The absorbance was measured at 415 nm with a spectrophotometer. All values were recorded as the mean ± SD in triplicates and expressed as mg CE/g [64].

#### 3.7.4. Determination of DPPH (2,2-Diphenyl-1-picrylhydrazyl) Radical Scavenging Activity

The antioxidant activity (DPPH Assay) of flour blends and tortillas was assessed using the method of Anyasi et al. [63]. Briefly, 0.2 g of samples was weighed and mixed with 2 mL of methanol and was incubated for a period of 30 min (25 ± 2 °C) before being centrifuged at 6000 rpm for a minimum of 10 min at 4 °C. Different concentrations of the samples (10, 20, 30, 40 and 50 mg/mL) were used to evaluate the IC50 of the sample which is the minimum amount of antioxidant needed to decrease the first DPPH absorbance with 50%. The value of IC50 was obtained by plotting disappearance percentage of DPPH as a function of concentration of the sample. A sample of 0.28 mL together with 0.25 mL DPPH were placed in a microplate and was covered by aluminium foil before it was incubated for 1 h at 25 ± 2°C. UV spectrophotometer microplate reader was used to read the absorbance at 517 nm. A standard used was gallic acid and results were expressed as percentage inhibition of the DPPH radical.

#### 3.7.5. Determination of Ferric Reducing Antioxidant Power (FRAP)

The method of Oyaizu [65] was followed to determine the FRAP of samples. A volume of about 100 µl of acidified methanol extract was adjusted to 1 mL with methanol in a test tube. About 2.5 mL of one percent potassium ferricyanide and 0.2 M phosphate buffer (pH 6.6) were added to the tube and vigorously shaken. A water bath was used to keep the mixture for 20 min at 50 °C. Approximately 2.5 mL of 10% trichloroacetic acid was added to the mixture after incubation and centrifuged at 5000 rpm for 10 min. The mixture was allowed to rest for 30 min and 2.5 mL distilled water was added to 2.5 mL of the supernatant and 0.5 mL of 0.1% ferric chloride. Extracts were measured at an absorbance of 700 nm and results were expressed in mg gallic acid equivalents (GAE) per gram of sample.

### 3.8. Physical Analyses of Tortillas

#### 3.8.1. Texture Analysis of Tortillas

The textural profile of the tortillas was analysed using texture analyser TAXTplus (Stable Microsystems, Surey, UK) attached to a 50 kg load cell with different probes, following the method described by Bejosano et al. [66]. The texture of the tortillas was measured 25 min after baking and this was done to allow them to cool. The accessory was a bar with a stainless steel 1/2-in. sphere tip. Test conditions were 50 mm distance from the puncture base. The tortilla was placed between two perforated plates, leaving an area 40 mm in diameter and in the middle where the bar that made contact. The force applied was that which was necessary to cause the tortilla to break. Hardness, which is the peak force required to achieve 50% deformation, was determined as a ratio of force divided by thickness (g-force/mm). To calculate cohesiveness, the total positive work done in cycle 2 was divided by the same in cycle 1. The sample length of the tortillas in the second compression was defined as springiness. Hardness x cohesiveness x springiness were used to calculate the chewiness of the tortillas.

#### 3.8.2. Physical Characteristics

Tortillas’ diameter was determined by averaging two perpendicular measurements on each of the three tortillas for a total of six observations per treatment. A venier calliper was used to measure the average thickness (mm) and this was done by stacking twelve (12) tortillas on top of each other. The digital weight balance was used to measure the weight of the tortillas. The method of Winstone [3] was used to calculate the spread ratio of the tortillas whereby the value was obtained by dividing the average value of diameter with average value of thickness of tortillas.

#### 3.8.3. Quality Properties

##### Puffing Degree

The method described by Cuevas-Martínez et al. [67] was adopted whereby scores of 1 to 3 were used to subjectively evaluate the puffing degree of the tortillas, where 1 (0–25%) shows little or no puffing, 2 (25% to 75%), medium puffing and 3 (75% to 100%)—complete puffing.

##### Rollability

The method of Cepeda et al. [68] was used to measure the rollability of the tortillas. Rollability is used to measure the degree of breakage of tortillas, whereby a scale from 1 to 5 was used with 1—unrollable/breaks, 2—has large tears, 3—shows many small cracks, 4—has a few small cracks and 5—rolls easily without cracking. A dowel with a diameter of 1 cm was used to evaluate each tortilla after being rolled around it.

### 3.9. Statistical Analysis

All measurements were performed in triplicate and results obtained were expressed as mean standard error deviation. The analysis of variance (ANOVA) was carried out using SPSS version 24 (IBM, Chicago, IL, USA). The mean was separated using the Duncan multiple range tests, *p* < 0.05 which was considered statistically significant.

## 4. Conclusions

The results showed that thermal properties (onset and peak temperature and enthalpy of gelatinisation) of composite flour samples were higher than those of the control flour. However, the end temperature values decreased. Proximate compositions of both maize flour and tortillas were improved by the addition of BGN flour and there was a significant increase on the protein content of both flour samples and tortillas. Polyphenolic compounds and antioxidant activity of flour samples and tortillas incorporated with BGN flour showed an increase. In terms of the texture, the control tortillas had higher hardness, springiness, gumminess, cohesiveness and chewiness values than the tortillas containing BGN flour. Weight and thickness of tortillas added with BGN flour were higher than control tortillas. However, diameter and spread ratio decreased. The puffing degree and rollability increased as more BGN flour was added to the formulation and full puffing of the tortillas was observed in the BGN_4_ sample. The study concluded that the substitution of maize with BGN flour produced tortillas with improved nutritional characteristics and better degree of puffing and rollability.

## Figures and Tables

**Table 1:** Thermal properties of control and maize-Bambara groundnut flour blends.

Sample	T_o_ (°C)	T_p_ (°C)	T_c_ (°C)	∆H (J/G)
Control	57.50 ± 0.58 ^a^	74.94 ± 0.61 ^a^	91.58 ± 1.49 ^b^	5.57 ± 1.39 ^a^
BGN_1_	61.70 ± 0.72 ^b^	75.86 ± 0.87 ^a^	89.93 ± 1.86 ^b^	6.31 ± 0.83 ^b^
BGN_2_	66.10 ± 0.70 ^c^	76.74 ± 0.71 ^ab^	87.70 ± 0.81 ^c^	8.59 ± 1.99 ^c^
BGN_3_	67.38 ± 0.44 ^d^	77.95 ± 0.32 ^b^	84.34 ± 0.45 ^a^	11.31 ± 0.29 ^d^
BGN_4_	71.95 ± 0.62 ^e^	78.45 ± 0.70 ^b^	81.72 ± 0.69 ^a^	14.81 ± 0.49 ^e^

BGN_1_, BGN_2_, BGN_3_ and BGN_4_ refer to the composite flours prepared from Bambara groundnut and maize flour (BGN: maize) in the percentages of 5:95, 10:90, 15:85 and 20:80 and maize flour was used as control. Values are mean ± standard deviation of triplicate determinations. Values followed by the same superscript(s) within the same column are not significantly different at *p* < 0.05. T_o_ = Onset temperature, T_p_ = Peak temperature, T_c_ = End temperature ΔH = enthalpy of gelatinisation.

**Table 2:** Proximate compositions of composite flours and tortillas per 100 g (dry basis).

Proximate Composition	Moisture	Ash	Protein	Fat	Fibre	Carbohydrates
**Flours**						
Control	3.69 ± 0.04 ^a^	1.23 ± 0.03 ^a^	3.31 ± 0.06 ^a^	1.74 ± 0.02 ^a^	2.03 ± 0.04 ^a^	88.00 ± 0.12 ^g^
BGN_1_	3.92 ± 0.08 ^b^	1.32 ± 0.03 ^b^	4.69 ± 0.02 ^b^	1.79 ± 0.03 ^a^	2.15 ± 0.04 ^ab^	86.00 ± 0.04 ^g^
BGN_2_	3.97 ± 0.05 ^b^	2.06 ± 0.02 ^e^	5.79 ± 0.05 ^c^	2.33 ± 0.04 ^b^	2.34 ± 0.05 ^b^	83.51 ± 0.01 ^f^
BGN_3_	4.12 ± 0.01 ^b^	2.16 ± 0.01 ^f^	7.24 ± 0.03 ^e^	2.40 ± 0.02 ^b^	2.77 ± 0.03 ^c^	81.31 ± 0.05 ^f^
BGN_4_	4.39 ± 0.21 ^c^	3.08 ± 0.05 ^g^	7.87 ± 0.03 ^f^	2.95 ± 0.01 ^c^	3.53 ± 0.26 ^e^	78.18 ± 0.11 ^e^
**Tortillas**						
Control	14.93 ± 0.13 ^d^	1.04 ± 0.04 ^a^	3.14 ± 0.02 ^a^	1.73 ± 0.02 ^a^	2.09 ± 0.03 ^a^	77.07 ± 0.27 ^e^
BGN_1_	20.59 ± 0.46 ^e^	1.33 ± 0.04 ^b^	4.60 ± 0.11 ^b^	1.78 ± 0.03 ^b^	2.24 ± 0.01 ^a^	69.46 ± 0.09 ^d^
BGN_2_	23.76 ± 0.07 ^f^	1.36 ± 0.04 ^b^	5.06 ± 0.01 ^c^	2.33 ± 0.04 ^b^	2.99 ± 0.57 ^d^	64.5 ± 0.56 ^c^
BGN_3_	25.04 ± 0.27 ^g^	1.53 ± 0.01 ^c^	5.41 ± 0.01 ^c^	2.40 ± 0.12 ^b^	3.84 ± 0.05 ^f^	61.78 ± 0.09 ^b^
BGN_4_	29.86 ± 0.25 ^h^	1.63 ± 0.04 ^d^	6.33 ± 0.52 ^d^	2.93 ± 0.04 ^c^	4.03 ± 0.02 ^g^	55.22 ± 0.58 ^a^

BGN_1_, BGN_2_, BGN_3_ and BGN_4_ refer to the composite flours prepared from Bambara groundnut and maize flour (BGN: maize) in the percentages of 5:95, 10:90, 15:85 and 20:80 and maize flour was used as control. Values are mean ± standard deviation of triplicate determinations. Values followed by the same superscript(s) within the column are not significantly different at *p* < 0.05.

**Table 3:** Polyphenolic compounds and antioxidant activity of composite flours and tortillas.

Sample	TPC (mg GAE/g)	TFC (mg CE/g)	FRAP (mg GAE/g)	DPPH (%)
**Flours**				
Control	43.82 ± 2.00 ^a^	10.83 ± 1.10 ^a^	0.071 ± 0.01 ^a^	43.09 ± 1.92 ^a^
BGN_1_	55.05 ± 3.01 ^b^	13.21 ± 1.23 ^b^	0.112 ± 0.01 ^b^	55.87 ± 3.07 ^b^
BGN_2_	60.21 ± 3.52 ^d^	17.34 ± 1.50 ^c^	0.124 ± 0.01 ^b^	65.46 ± 3.48 ^d^
BGN_3_	66.04 ± 3.88 ^e^	20.44 ± 1.68 ^d^	0.141 ± 0.03 ^c^	75.19 ± 5.60 ^e^
BGN_4_	72.05 ± 4.22 ^f^	23.58 ± 1.98 ^e^	0.156 ± 0.04 ^d^	80.54 ± 6.17 ^f^
**Tortillas**				
Control	46.61 ± 2.10 ^b^	10.03 ± 1.15 ^a^	0.106 ± 0.01 ^b^	60.70 ± 3.50 ^c^
BGN_1_	60.11 ± 3.49 ^d^	10.87 ± 1.30 ^a^	0.118 ± 0.02 ^b^	77.87 ± 4.96 ^b^
BGN_2_	62.11 ± 3.92 ^d^	13.31 ± 1.63 ^b^	0.126 ± 0.02 ^b^	82.38 ± 5.93 ^f^
BGN_3_	72.86 ± 4.20 ^f^	16.43 ± 1.70 ^c^	0.131 ± 0.02 ^c^	87.21 ± 6.32 ^g^
BGN_4_	79.31 ± 5.60 ^g^	19.87 ± 1.99 ^d^	0.183 ± 0.01 ^d^	90.99 ± 6.66 ^h^

BGN_1_, BGN_2_, BGN_3_ and BGN_4_ refer to the composite flours and tortillas prepared from Bambara groundnut and maize flour (BGN: maize) in the percentages of 5:95, 10:90, 15:85 and 20:80 and maize flour was used as control. TPC = total phenolic content, TFC = total flavonoids content. Values are mean ± standard deviation of triplicate determinations. Values followed by the same superscript(s) within the same column are not significantly different at *p* < 0.05.

**Table 4:** Textural profile analysis of tortillas added with Bambara groundnut flour.

Sample	Hardness (N)	Springiness (mm)	Cohesiveness	Gumminess	Chewiness
Control	8.99 ± 1.55 ^e^	0.58 ± 0.003 ^e^	0.09 ± 0.003 ^d^	24.17 ± 2.69 ^e^	13.97 ± 1.22 ^d^
BGN_1_	8.82 ± 1.33 ^de^	0.57 ± 0.004 ^d^	0.06 ± 0.002 ^c^	20.96 ± 2.20 ^d^	11.79 ± 1.12 ^d^
BGN_2_	8.52 ± 1.20 ^c^	0.56 ± 0.004 ^c^	0.03 ± 0.001 ^b^	15.05 ± 1.70 ^c^	8.41 ± 0.99 ^c^
BGN_3_	7.72 ± 1.05 ^b^	0.54 ± 0.004 ^b^	0.03 ± 0.001 ^ab^	13.24 ± 1.32 ^b^	7.17 ± 0.89 ^b^
BGN_4_	6.52 ± 1.00 ^a^	0.52 ± 0.007 ^a^	0.02 ± 0.001 ^a^	11.81 ± 1.06 ^a^	6.12 ± 0.80 ^a^

BGN_1_, BGN_2_, BGN_3_ and BGN_4_ refer to the tortillas prepared from Bambara groundnut and maize flour (BGN: maize) in the percentages of 5:95, 10:90, 15:85 and 20:80 and maize flour was used as control. Values are mean ± standard deviation of triplicate determinations. Values followed by the same superscript(s) within the same column are not significantly different at *p* < 0.05.

**Table 5:** Physical characteristics of tortillas added with Bambara groundnut flour.

Sample	Weight (g)	Diameter (cm)	Thickness (mm)	Spread Ratio
Control	35.93 ^a^ ± 0.70 ^a^	14.60 ± 0.10 ^e^	2.17 ± 0.58 ^a^	6.72 ± 0.16 ^e^
BGN_1_	36.92 ^b^ ± 0.12 ^b^	14.17 ± 0.58 ^d^	2.37 ± 0.58 ^b^	5.99 ± 0.16 ^d^
BGN_2_	37.67 ^c^ ± 0.24 ^c^	13.80 ± 0.10 ^c^	2.60 ± 0.10 ^c^	5.31 ± 0.25 ^c^
BGN_3_	38.25 ^d^ ± 0.29 ^d^	13.40 ± 0.10 ^b^	2.83 ± 0.58 ^d^	4.72 ± 012 ^b^
BGN_4_	39.26 ^e^ ± 0.19 ^e^	13.07 ± 0.15 ^a^	3.07 ± 0.34 ^e^	4.17 ± 0.25 ^a^

BGN_1_, BGN_2_, BGN_3_ and BGN_4_ refer to the tortillas prepared from Bambara groundnut and maize flour (BGN: maize) in the percentages of 5:95, 10:90, 15:85 and 20:80 and maize flour was used as control. Values are mean ± standard deviation of triplicate determinations. Values followed by the same superscript(s) within the same column are not significantly different at *p* < 0.05.
